# Prevalence and clinical significance of potential drug-drug interactions among lung transplant patients

**DOI:** 10.3389/fphar.2024.1308260

**Published:** 2024-02-06

**Authors:** Jiali Zhang, Danyi Ma, Meng Chen, Yanting Hu, Xveying Chen, Jingyu Chen, Man Huang, Haibin Dai

**Affiliations:** ^1^ Department of Pharmacy, Second Affiliated Hospital, Zhejiang University School of Medicine, Hangzhou, China; ^2^ Department of General Intensive Care Unit, Second Affiliated Hospital, Zhejiang University School of Medicine, Hangzhou, China; ^3^ Department of Lung Transplantation, Second Affiliated Hospital, Zhejiang University School of Medicine, Hangzhou, China

**Keywords:** potential drug-drug interactions, lung transplant, intensive care unit, tacrolimus, voriconazole

## Abstract

**Background:** Drug-drug interactions (DDIs) are a major but preventable cause of adverse drug reactions. There is insufficient information regarding DDIs in lung transplant recipients.

**Objective:** This study aimed to determine the prevalence of potential DDIs (pDDIs) in intensive care unit (ICU) lung transplant recipients, identify the real DDIs and the most frequently implicated medications in this vulnerable population, and determine the risk factors associated with pDDIs.

**Methods:** This retrospective cross-sectional study included lung transplant recipients from January 2018 to December 2021. Pertinent information was retrieved from medical records. All prescribed medications were screened for pDDIs using the Lexicomp^®^ drug interaction software. According to this interaction software, pDDIs were classified as C, D, or X (C = monitor therapy, D = consider therapy modification, X = avoid combination). The Drug Interaction Probability Scale was used to determine the causation of DDIs. All statistical analysis was performed in SPSS version 26.0.

**Results:** 114 patients were qualified for pDDI analysis, and total pDDIs were 4051. The most common type of pDDIs was category C (3323; 82.0%), followed by D (653; 16.1%) and X (75; 1.9%). Voriconazole and posaconazole were the antifungal medicine with the most genuine DDIs. Mean tacrolimus concentration/dose (Tac C/D) before or after co-therapy was considerably lower than the Tac C/D during voriconazole or posaconazole co-therapy (*p* < 0.001, *p* = 0.027). Real DDIs caused adverse drug events (ADEs) in 20 patients. Multivariable logistic regression analyses found the number of drugs per patient (OR, 1.095; 95% CI, 1.048–1.145; *p* < 0.001) and the Acute Physiology and Chronic Health Evaluation II (APACHE Ⅱ) score (OR, 1.097; 95% CI, 1.021–1.179; *p* = 0.012) as independent risk factors predicting category X pDDIs.

**Conclusion:** This study revealed a high incidence of both potential and real DDIs in ICU lung transplant recipients. Immunosuppressive drugs administered with azole had a high risk of causing clinically significant interactions. The number of co-administered drugs and APACHE Ⅱ score were associated with an increased risk of category × drug interactions. Close monitoring of clinical and laboratory parameters is essential for ensuring successful lung transplantation and preventing adverse drug events associated with DDIs.

## Introduction

Lung transplantation has grown into a life-saving treatment with enhanced quality of life for patients suffering from end-stage respiratory failure unresponsive to conventional medicinal or surgical therapies (Kotloff and Thabut, 2011; Adegunsoye et al., 2017). The number of transplants performed has continuously increased over the years. According to the International Society for Heart and Lung Transplantation (ISHLT), almost 70,000 adult lung transplants were performed globally in 2018 (Chambers et al., 2021). Lung transplant recipients are treated with potent immunosuppressants to prevent graft rejection. Commonly recommended medications include tacrolimus (Tac), cyclosporine, mycophenolate mofetil, and corticosteroids (Sweet, 2013). Due to various comorbid diseases, including diabetes, cardiovascular disease, and cerebral vascular disease, transplant patients additionally receive multiple drugs.

As known, a potential drug-drug interaction (pDDI) can be defined as prescription of one drug affects another drug’s activity being used simultaneously, irrespective of adverse events (Magro et al., 2012). Intensive care unit (ICU) patients receive the drug prescriptions twice as many drugs compared to non-ICU patients (Cullen et al., 1997). Due to polypharmacotherapy, ICU lung transplant recipients are at a greater risk for pDDIs. Especially pDDIs involving immunosuppressive drugs with a limited therapeutic index are particularly susceptible to adverse drug events (ADEs) (Gago-Sánchez et al., 2021; Dewey et al., 2023). The tendency is complicated by illness severity and organ failure, which can alter medication pharmacologic response.

Although potential DDIs are very common, the clinical consequences vary widely, and ADEs rarely occur (Leal Rodríguez et al., 2022). Therefore, real drug-drug interactions which are defined as DDIs associated with ADE-related hospital admissions, ADEs that were present at hospital admissions and laboratory deviations have more clinical relevance (Gago-Sánchez et al., 2021; Očovská et al., 2023). It is a significant cause of adverse drug effects and a public health concern. A 2007 study evaluating the effects of DDIs estimated that they were responsible for approximately 0.054% of emergency room visits, 0.57% of all hospital admissions and 0.12% of rehospitalizations (Becker et al., 2007). DDIs also impose a major fiscal burden on the healthcare system (Shad and Marsh, 2001). Since pDDIs are mostly preventable, and minimizing them can potentially reduce morbidity and mortality, research is essential to identify pDDIs in a specific treatment environment and assess approaches to mitigate them. Interactions are crucial to transplant pharmacotherapy because of their clinical significance and frequency. However, multiple studies have evaluated pDDIs in post-transplant patients. In published researches of bone marrow transplant patients, for instance, the proportion of potential clinically relevant interactions ranged from 21.4% to 82.5% (Guastaldi et al., 2011; Trevisan et al., 2015). Julia Amkreutz et al. reported 99 serious potential DDIs among kidney transplant patients per 100 patient days (Amkreutz et al., 2017). So far, no studies have observed the prevalence of pDDIs in ICU for lung transplant recipients and evaluated their clinical impact. This study seeks to fill this gap and extract key insight for clinicians to mitigate this issue.

Hence, the primary goals of this study were to evaluate the occurrence and characteristics of pDDIs and real DDIs in ICU lung transplant recipients after their initial lung transplantation, identify the most frequently implicated medications in this vulnerable population, and determine the risk factors associated with DDIs in lung transplantation.

## Materials and methods

### Study design and setting

Retrospective cross-sectional research was carried out at the Second Affiliated Hospital of Zhejiang University, School of Medicine (SAHZU), an eastern China-based tertiary care hospital with 3,200 licensed beds where liver, kidney, bone marrow, lung, and heart transplants are carried out.

All patients with respiratory diseases who underwent lung transplantation for the first time from January 2018 to December 2021 were included in this study if they were prescribed at least two drugs during their ICU stay. Post-transplant ICU patients with missing medication data were excluded from the research.

### Data collection and definition

For every patient, demographic and clinical data from electronic medical records were retrieved and entered into a structured data collection form encompassing the following details: gender, age, major diagnosis, ICU stay duration, Body Mass Index (BMI), primary disease (e.g., pulmonary hypertension, interstitial lung disease, bronchiectasis, chronic obstructive pulmonary disease, pulmonary infection, and silicosis), comorbidities (including diabetes mellitus, hypertension, malignancy, cerebrovascular accident, and infectious disease), and the Acute Physiology and Chronic Health Evaluation II (APACHE II) score. To estimate disease severity, APACHE II employed a point score based on the initial values of 12 routine physiologic parameters, age, and previous health status to determine the disease severity (Knaus et al., 1985). If the patient received Tac and voriconazole or posaconazole concomitantly, the Tac whole blood trough concentrations and dose were recorded. All prescribed medications were examined using the Lexicomp^®^ drug interaction software (Wolters Kluwer Health, Inc. Riverwoods, IL., United States of America) for potential DDIs. This is a copyrighted drug-drug, drug-herb, and herb-herb analysis tool, provided by UpToDate^®^ utilizing Lexicomp^®^ clinical content. We utilized the Second Affiliated Hospital of Zhejiang University, School of Medicine’s Library E-Resources to access Lexicomp^®^ drug interactions via UpToDate^®^ (Abbas et al., 2022). The identified interactions were categorized as category X, indicating the need to avoid the combination; D, requiring therapy modification; and C, suggesting therapy monitoring following the Lexicomp criteria. If the same DDI alert occurred for the same patient multiple times during hospitalization, it was considered only once.

We collected information on all medicines administered to eligible patients until they are discharged from ICU. The pharmacists used electronic assisted prescribing to review every prescription line, and any pDDIs found were entered in pairs. To assess whether these pDDIs could have a real clinical impact, the pharmacists conducted a thorough review of each patient considering all clinical information. Laboratory tests and/or patient symptoms confirmed clinically manifested DDIs. The real DDI was discovered when it induced ADEs. If the pharmacist and the physician agreed on an adverse patient outcome, the Drug Interaction Probability Scale (DIPS) tool was used to assess the likelihood of a causal relationship between a pDDI and an event; those with a probability of 5–8 points or more were considered real DDIs (Horn et al., 2007).

Because they are less likely than other pharmaceuticals to produce systemic drug interactions, the following medicines were excluded: 1) inhaled medications, such as salbutamol, ipratropium, budesonide, acetylcysteine, and epinephrine; 2) topical medications; 3) vaccines; and 4) any drug administered as needed and irregularly and intermittently, including diuretics, antipyretics, and analgesics (drugs not taken on consecutive days or at regular intervals). Concurrent exposure was regarded to occur when medications were administered within 24 h. But switching one medication for another was not considered coadministration. During the study period, 24 drugs were unsearchable in the Lexicomp^®^ drug interaction software. Consequently, these drugs were excluded (Supplementary Appendix SA1).

### Data analysis and statistics

For continuous variables, descriptive statistics were expressed as median (interquartile range [IQR]) or mean ± standard deviation (SD) depending on whether they are normally distributed. Categorical variables were described with frequency and percentage. Continuous variables were compared using Student’s t-tests for independent variables and the Mann–Whitney *U* test for non-normally distributed data. Pearson’s χ^2^-test was used to compare categorical variables. To assess the potential association between the occurrence of category X DDIs, separate logistic regression analyses and multivariable logistic regression analyses were conducted to investigate risk factors linked with identified pDDIs. The odds ratio (OR) and 95% confidence interval (CI) were calculated for each variable. Covariates were determined using significant variables from the univariable model (*p* < 0.2). All statistical assessments were conducted using descriptive statistics from the SPSS software for Windows version 26.0. Results with *p* values <0.05 were deemed statistically significant. Figures were generated using SigmaPlot software for Windows version 14.0.

## Results

**FIGURE 1:**
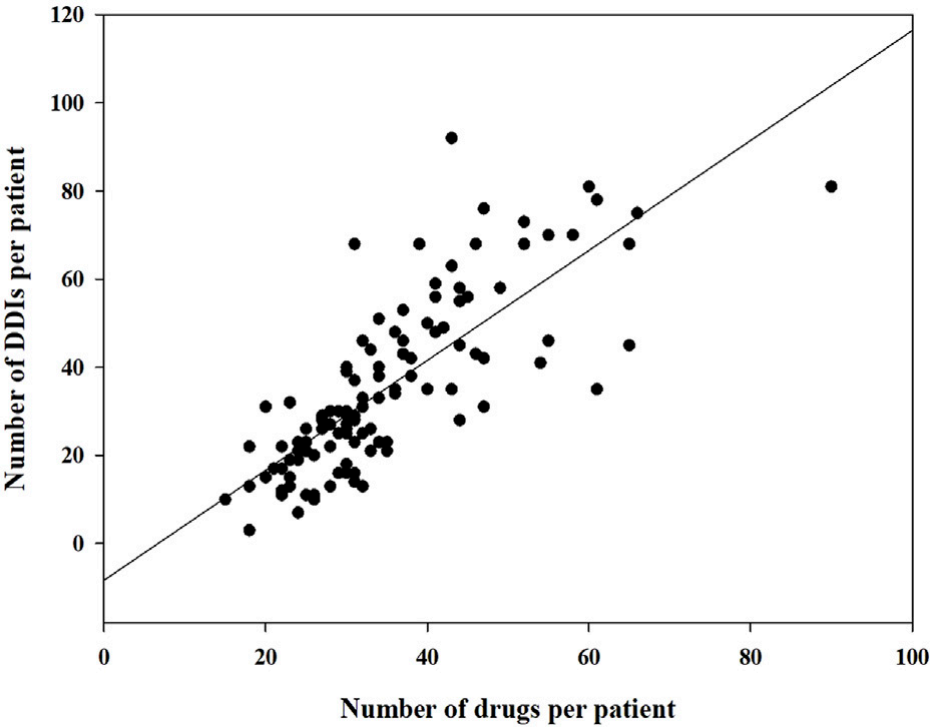
Correlation between the number of drugs prescribed and the number of drugs prescribed and the number of drug-drug interactions detected.

During this study, all lung transplant recipients received more than two drugs during their ICU stay, no patients were excluded. Finally, 114 eligible patients were enrolled for pDDI analysis. Their mean age was 54.54 ± 15.16 years old. The male percentage was predominant in the study population, accounting for 81.6%, and 28.4% were females. Interstitial lung disease (53.5%) was the most frequent diagnosis for lung transplantation. Infectious disease (74.6%) was the most frequent comorbid condition. The patient severity, measured using the APACHE Ⅱ score, was 10, with an interquartile range of 7–15. The median length of stay in the ICU interquartile was 20 days, with a range of 11–31 days. The 114 patients were prescribed and administered a median of 32 medications (interquartile range, 27–41) from various drug categories. Table 1 presents the clinical and demographic details of the cohort. Finally, the study observed a total of 4051pDDIs, as shown in Table 2. The number of potential DDIs per patient interquartile ranged from 21 to 47, with a median of 30. Figure 1 illustrates a positive correlation between the detected pDDIs and the number of drugs prescribed. Among the 4051 identified pDDIs, the prevailing type was category C (3323; 82.0%), followed by D (653; 16.1%) and X (75; 1.9%). Table 3 and Table 4 not only present the ten most common Category X and Category D interactions, but also list real DDIs in this study. Real DDIs were detected in 20 patients, and the prevalence was 17.54%. Of the 9 pDDIs amikacin-polymyxin B pair, 2 (22.22%) were real DDIs. As shown in Table 3, the two patients both experienced nephrotoxic.

**TABLE 1:** Demographic and clinical characteristics of the patients included (n = 114).

Characteristics	n (%) or median (IQR)
Age, years	54.54 ± 15.16
Gender, male (%)	93 (81.6%)
Gender, female (%)	21 (28.4%)
BMI, kg/m^2^	20.35 ± 4.54
APACHE Ⅱ score	10 (7,15)
Length of ICU stay, days	20 (11,31)
Primary disease (%)	
Pulmonary hypertension	32 (28.1%)
Interstitial lung disease	61 (53.5%)
Bronchiectasis	11 (9.6%)
Chronic obstructive pulmonary disease	32 (28.1%)
Pulmonary infection	36 (31.6%)
Silicosis	19 (16.7%)
Comorbidities (%)	
Diabetes mellitus	21 (18.4%)
Hypertension	21 (18.4%)
Malignancy	5 (4.4%)
Cerebrovascular accident	4 (3.5%)
Infectious disease	85 (74.6%)
Past drug allergy (%)	
Yes	24 (21.1%)
No	90 (78.9%)
Number of drugs per patient	32 (27,41)
Number of DDIs per patient	30 (21,47)

Data are presented as n (%) or mean ± SD; BMI, body mass index.

**TABLE 2:** Numbers of potential DDIs detected.

Category of DDIs	Numbers of DDIs detected	%	
X	75	1.9	The combination should be avoided
D	653	16.1	The combination required modification
C	3323	82.0	The combination suggested monitoring
Total	4051	100.0	

**TABLE 3:** Potential effects, frequency of the 10 most prevalent class X drug-drug interactions and real drug-drug interactions in the study population.

Class	Drug pair	Real DDI DIPS score (causal relationship)	Frequency (number of real DDI)	Potential effects	Time (days) to develop ADEs after drug combination	Management
X	Amikacin + Polymyxin B	5	9 (2)	Increase risk of nephrotoxic	5.50 ± 2.12 (4–7)	Monitor renal function, reduce dose of amikacin or change antibiotics
	Cyclosporine + Spironolactone	-	5 (0)	enhance the hyperkalemic effect	-	-
	Amiodarone + Levofloxacin	NA	5 (NA)	enhance the QTc-prolonging effect	NA	NA
	Atorvastatin + Posaconazole	5	5 (2)	Increase risk of myopathy/rhabdomyolysis and hepatic failure	22.5 ± 12.0 (14–31)	Interrupt atorvastatin
	Voriconazole + Amiodarone	NA	3 (NA)	enhance the QTc-prolonging effect	NA	NA
	Sertraline + Linezolid	-	3 (0)	enhance the serotonergic effect	-	-
	Atorvastatin + Cyclosporine	-	3 (0)	Increased risk of myopathy/rhabdomyolysis and renal failure	-	-
	Rivaroxaban + Enoxaparin	-	3 (0)	enhance the anticoagulant effect	-	-
	Alfacalcidol + Vitamin D_3_	NA	3 (NA)	enhance the adverse effect of Vitamin D Analogs	NA	NA
	Domperidone + Voriconazole	NA	2 (NA)	enhance the QTc-prolonging effect	NA	NA
	Amikacin + Mannitol	-	2 (0)	enhance the nephrotoxic effect	-	-
	Tacrolimus + Foscarnet	-	2 (0)	enhance the nephrotoxic effect	-	-
	Morphine + Linezolid	-	2 (0)	enhance the adverse effect of Morphine	-	-
	Calcitriol + Vitamin D_3_	-	2 (0)	enhance the adverse effect of Vitamin D Analogs	-	-
	Escitalopram + Linezolid	-	2 (0)	Increase risk of serotonin syndrome	-	-

DIPS, drug interaction probability scale; NA, not applicable (NA, is used when data cannot be collected).

**TABLE 4:** Potential effects, frequency of the 10 most prevalent class D drug-drug interactions and real drug-drug interactions in the study population.

Class	Drug pair	Real DDI DIPS score (causal relationship)	Frequency (number of real DDI)	Potential effects	Time (days) to develop ADEs after drug combination	Management
D	Remifentanil + Propofol	NA	71 (NA)	enhance the CNS depressant effect	NA	NA
	Tacrolimus + Voriconazole	6	44 (9 renal, 4 liver)	increase the serum levels of Tac causing renal or hepatic dysfunction	6.2 ± 2.5 (3–9)	Reduce dose of Tac, monitor Tac concentrations, renal function and liver function
	Remifentanil + Linezolid	-	30 (0)	Increase risk of opioid toxicity and enhance the serotonergic effect	-	-
	Noradrenaline + Linezolid	-	30 (0)	enhance the hypertensive effect of Sympathomimetics	-	-
	Remifentanil + Pregabalin	NA	29 (NA)	enhance the CNS depressant effect	NA	NA
	Remifentanil + Midazolam	NA	24 (NA)	enhance the CNS depressant effect	NA	NA
	Prednisone + Sodium Bicarbonate	NA	20 (NA)	decrease the bioavailability of Corticosteroids	NA	NA
	Prednisone + Calcium Carbonate	NA	18 (NA)	decrease the bioavailability of Corticosteroids	NA	NA
	Remifentanil + Gabapentin	NA	16 (NA)	enhance the CNS depressant effect	NA	NA
	Tacrolimus + Posaconazole	6	15 (2 liver, 1 renal)	increase the serum concentration of Tac	14.7 ± 9.3 (7–25)	Reduce dose of Tac, monitor Tac concentrations, renal function and liver function

DIPS, drug interaction probability scale; CNS, central nervous system; NA, not applicable (NA, is used when data cannot be collected), Tac, tacrolimus.

**FIGURE 2:**
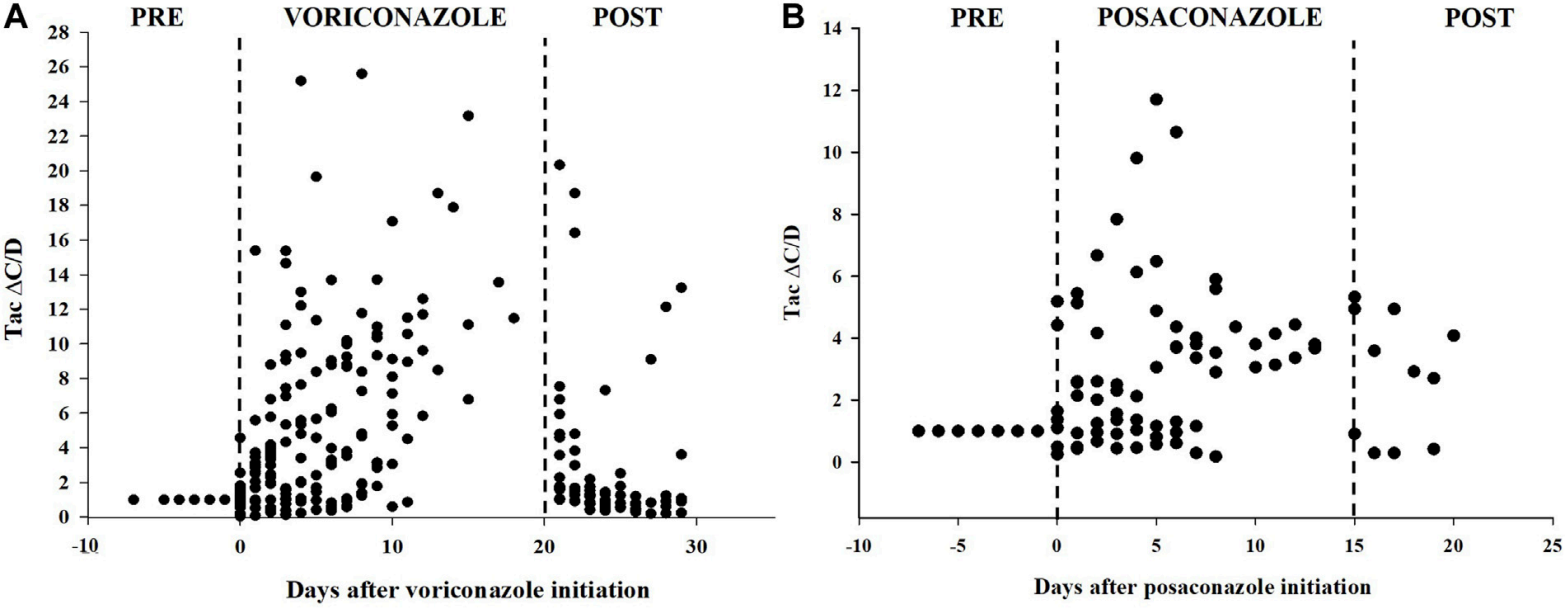
Tacrolimus dose-corrected concentrations over time in patients treated with voriconazole **(A)** or posaconazole **(B)**. Delta of dose-corrected concentrations (∆C/D) are plotted as a ratio (fold increase) *versus* the average C/D value before triazole therapy, which is therefore set to 1.

Voriconazole and posaconazole were the antifungal drug with the most genuine DDIs (Gago-Sánchez et al., 2021). Of the 44 pDDIs voriconazole-Tac pair, 13 (25.55%) were real DDIs, and of the 15 pDDIs posaconazole-Tac pair, 3 (20%) were real DDIs. Among lung recipients, a distinct analysis was conducted on the subgroup of patients whose baseline Tac C/D was obtained prior to (rather than after) voriconazole or posaconazole administration or who had at least one Tac C/D available after voriconazole or posaconazole initiation. A total of 44 patients were recruited in voriconazole subgroup, meanwhile 15 patients in posaconazole subgroup. Ten patients in voriconazole subgroup and four patients in posaconazole subgroup, however, were later excluded because they satisfied the exclusion criteria. In these patients, the mean Tac C/D was 4.86 ± 6.35 ng/μL/mg before or after co-treatment, which was significantly lower than the Tac C/D (10.62 ± 10.57 ng/μL/mg) during voriconazole therapy (*p* < 0.001). Simultaneously, the mean Tac C/D before or after co-treatment was also significantly lower than the Tac C/D during posaconazole therapy (*p* = 0.027). The average concentration and dose of Tac were both significant differences between the two groups for voriconazole (*p* < 0.001) (Table 5). Figure 2 depicts the time-related change in Tac ∆C/D during voriconazole or posaconazole therapy in patients with a mean pre-azole Tac C/D available.

**TABLE 5:** Tac concentration/dose between baseline and period of azole co-therapy.

Triazole		Before/after co-therapy	During azole co-therapy	*p*
Voriconazole	Tac concentration (ng/μL)	5.31 ± 3.06	8.37 ± 4.39	<0.001
	Tac dose (mg/day)	2.86 ± 2.25	1.46 ± 1.19	<0.001
	Tac C/D (ng/μL/mg)	4.86 ± 6.35	10.62 ± 10.57	<0.001
Posaconazole	Tac concentration (ng/μL)	6.01 ± 2.79	7.63 ± 3.64	0.021
	Tac dose (mg/day)	3.21 ± 2.44	2.55 ± 2.17	0.356
	Tac C/D (ng/μL/mg)	3.36 ± 3.53	5.30 ± 4.42	0.027

C/D, dose-corrected through concentration; Tac, tacrolimus.

Table 6 and Table 7 present the results of the univariable and multivariate logistic regression analysis models used to analyze the risk factors associated with category X drug-drug interaction. Univariable analysis showed a statistically significant correlation between the number of category X drug-drug interactions and APACHE Ⅱ score (*p* < 0.001), length of ICU stay (*p* < 0.001), number of drugs per patient (*p* < 0.001) and number of DDIs per patient (*p* < 0.001). There was no significant relationship with age, gender, BMI, primary disease, or past drug allergy. Multivariable logistic regression analyses showed that the number of drugs per patient (OR, 1.095; 95% CI, 1.048–1.145; *p* < 0.001) and APACHE Ⅱ score (OR, 1.097; 95% CI, 1.021–1.179; *p* = 0.012) added statistically significantly to predict category X drug-drug interactions (Table 7).

**TABLE 6:** Logistic regression analysis for factors associated with class X drug-drug interactions in the study population.

Variable	With X (n = 48)	Without X (n = 66)	*p*
Age, years	54.81 ± 17.18	54.33 ± 13.65	0.869
Age, ≥ 65	35 (72.9%)	54 (81.8%)	0.257
Gender, male (%)	38 (79.2%)	55 (83.3%)	0.571
Gender, female (%)	10 (20.8%)	11 (16.7%)	
BMI, kg/m^2^	21.09 ± 4.58	19.80 ± 4.48	0.135
APACHE Ⅱ score	12 (10,19)	10 (6,12)	<0.001
Length of ICU stay, days	26 (15,36)	7 (14,26)	<0.001
Primary disease (%)			
Pulmonary hypertension	10 (20.8%)	22 (33.3%)	0.143
Interstitial lung disease	25 (52.1%)	36 (54.5%)	0.795
Bronchiectasis	8 (16.7%)	3 (4.5%)	0.065
Chronic obstructive pulmonary disease	12 (25.0%)	20 (30.3%)	0.534
Pulmonary infection	17 (35.4%)	19 (28.8)	0.452
Silicosis	6 (12.8%)	13 (19.7%)	0.332
Comorbidities (%)			
Diabetes mellitus	8 (16.7%)	13 (19.7%)	0.680
Hypertension	12 (25.0%)	9 (13.6%)	0.122
Malignancy	4 (8.3%)	1 (1.5%)	0.196
Cerebrovascular accident	1 (2.1%)	3 (4.5%)	0.849
Infectious disease	41 (85.4%)	44 (66.7%)	0.023
Past drug allergy (%)			
Yes	13 (27.1%)	11 (16.7%)	0.178
No	35 (72.9%)	55 (83.3%)	
Number of drugs per patient	40 (32,47)	30 (25,34)	<0.001
Number of DDIs per patient	45 (30,67)	25 (16,34)	<0.001

**TABLE 7:** Univariable and multivariable logistic regression analysis for independent risk factors for class X drug-drug interactions in the study population.

Risk factor	Unadjusted OR (95% CI)	*p*	Adjusted OR (95% CI)	*p*
Number of drugs per patient	1.101 (1.053–1.151)	<0.001	1.095 (1.048–1.145)	<0.001
APACHE Ⅱ score	1.107 (1.036–1.183)	0.003	1.097 (1.021–1.179)	0.012

OR, odd ratio; 95% CI, 95% confidence interval.

## Discussion

Our research demonstrates a high prevalence of post-transplant pDDIs in ICU lung transplant recipients, and some detected DDIs may be clinically relevant, especially when administered with triazole. No published research has so far considered this problem in lung transplant recipients. In this study, all patients (100%) showed some pDDIs, and the prevalence of real DDIs was 17.54%. Our findings significantly indicated a greater incidence of pDDIs compared to other similar studies. Danilo D. et al. reported that 82.5% of patients undergoing hematopoietic stem cell transplantation were exposed to at least one significant and concomitantly contraindicated pDDI (Trevisan et al., 2015). On the other hand, Ana Isabel Gago-Sánchez et al. revealed that the prevalence of real DDIs among transplant recipients was merely 21.7% (Gago-Sánchez et al., 2021). We may be able to explain the high occurrence of pDDIs in light of this research. The primary factor that might have potentially contributed to this outcome is that lung transplantation is a complex disease that usually complicates multiple comorbidities (e.g., previous or new-onset diabetes mellitus, hypertension, malignancy, cerebrovascular accident, and infectious disease) that require different medications for treatment. The median of medicine *per capita* was 32 in our study group. DDIs were classified based on the Lexicomp^®^ drug interaction software, which is widely recognized among medical professionals and has been cited in several researches (Kheshti et al., 2016; Gago-Sánchez et al., 2021).

In the present study, category X and D potential drug interactions constituted 1.9% and 16.1%, respectively. However, the most common type of pDDI detected in our investigation was C. Although Type C pDDIs seldom result in major repercussions, they must be carefully monitored to minimize any potential negative effects. Infections occur commonly in lung transplant recipients and are usually treated with antimicrobial drugs or antiviral agents (Joean et al., 2022). Antimicrobial drug-induced nephrotoxicity has been recognized and studied for many years (Zhang et al., 2021; Roughead et al., 2022). In a study of 100 patients undergoing allogeneic hematopoietic stem cell transplantation, patients receiving two or more nephrotoxic anti-infective drugs were at least three times more likely to develop acute kidney injury (Günay et al., 2023). The class X DDI observed most frequently in our study was between amikacin and polymyxin B. Amikacin, an aminoglycoside, is known to cause nephrotoxicity and has a limited therapeutic range (Yamada et al., 2021). Polymyxin B, used in treating severe infections, is reported to have an incidence of nephrotoxicity ranging from 14.0% to 50.6% (Ouderkirk et al., 2003; Elias et al., 2010). Using them with concurrent nephrotoxic agents increases their adverse effects on the kidneys. In our study, Nephrotoxicity occurred in two of the nine patients treated with amikacin-polymyxin B. Antiviral medications have also been linked to nephrotoxicity (Leowattana, 2019). Amikacin + mannitol and tacrolimus + foscarnet were also identified in this research as potential interactions that interfere with nephrotoxicity.

It is important to highlight the connection between the occurrence of pDDIs and the use of medications that prolong the QT interval, as the risk of cardiotoxicity with these drugs is a developing concern (Haitao et al., 2022). The amiodarone + levofloxacin, voriconazole + amiodarone, and domperidone + voriconazole interactions observed in this study all have the potential to cause the aforementioned unwanted results. Amiodarone is a commonly used antiarrhythmic drug recognized for its QT interval-prolonging potential (Then et al., 2023). Due to the potential danger, administering amiodarone together with other medications that have QT-prolonging potential is contraindicated, even though multiple recent studies have been unable to show any significant risk (Van Der Sijs et al., 2009; Meid et al., 2017; Then et al., 2023). According to the literature, voriconazole and domperidone can cause QT interval prolongation too (Mourad et al., 2019).

Central nervous system (CNS) interactions are also a common type of interaction in lung transplant recipients. A conducted study in a general ICU revealed that 40% of the pDDIs were associated with drugs acting on CNS (Lima and Cassiani, 2009). Among the CNS interactions, common pDDI was between remifentanil and propofol in this study. A US Food and Drug Administration (FDA) drug safety communication states that combined use of opioids with benzodiazepines or other CNS depressant drugs can lead to serious adverse effects include difficulty breathing and death (US Food and Drug Administration, 2016). However, for critically ill patients receiving mechanical ventilation, this combination offers comfort and relief from anxiety. Thus, this interaction is used in intensive care with a therapeutic goal (Devlin and Roberts, 2009). Furthermore, CNS interactions associated with selective serotonin reuptake inhibitors (SSRIs) are mainly reported in depression patients (de Leon and Spina, 2018). Linezolid is a synthetic oxazolidinone antibiotic effective against resistant Gram-positive bacteria, capable of reversibly inhibiting monoamine oxidase (Phillips et al., 2015). Severe serotonin syndrome typically occurs with the concomitant administration of two or more serotonergic drugs, with the particular danger associated with the combination of serotonergic drugs and monoamine oxidase inhibitors (MAOIs) (Bijl, 2004; Buckley et al., 2014). Lung transplant patients often have multiple diseases and are susceptible to infection with Gram-positive bacteria (Påhlman et al., 2021). When lung transplant recipients use linezolid and SSRIs simultaneously, prescribers should be vigilant about this potential drug interaction.

In immunocompromised people, invasive aspergillosis is a major source of morbidity and mortality. (Shiraishi, 2016; Husain and Camargo, 2019). Between 4% and 23% of lung transplant recipients develop invasive aspergillosis (Husain et al., 2006). Voriconazole is the known drug of choice for invasive aspergillosis (Pappas et al., 2010; Neofytos et al., 2021). It inhibits cytochrome P450 3A4, a critical enzyme in metabolizing immunosuppressant drugs. Inhibiting CYP3A4 and P-glycoprotein increases immunosuppressant blood concentrations and adds to an increased risk of adverse outcomes due to severe immunosuppression and toxicity (e.g., nephrotoxicity, neurotoxicity) (Wada et al., 2020; Crone et al., 2022). The antifungal drugs' pDDI study revealed a prevalence of 71.4% (Guastaldi and Secoli, 2011). A narrow therapeutic index can result in clinically significant interactions with tacrolimus (Groll et al., 2017). In this study, all real DDIs related to antimycotics for systemic use were voriconazole or posaconazole. Of 44 patients treated with tacrolimus-voriconazole pair, 9 developed nephrotoxicity and 4 developed hepatotoxicity. Meanwhile, 2 individuals suffered hepatotoxicity and 1 acquired nephrotoxicity after receiving tacrolimus combined posaconazole. According to the reports, increased concentration of tacrolimus has been associated with ADEs (Tolou-Ghamari, 2012; Zhao et al., 2022). In the current analysis, voriconazole and posaconazole both significantly increased the tac concentration. However, the proportional increase in Tac ∆C/D resulting from voriconazole or posaconazole co-therapy varied substantially among the patients, consistent with prior observations in organ recipients (Mori et al., 2012; Vanhove et al., 2017). For instance, in a multicenter retrospective cohort analysis by T. Vanhove et al., among 100 solid organ recipients using tacrolimus-voriconazole or posaconazole co-therapy, suggested that Tac C/D increased by a factor 5.0 ± 2.7 (range 1.0–20.2) for voriconazole and 4.4 ± 2.6 (range 0.9–18.0) for posaconazole (Vanhove et al., 2017). Nonetheless, some studies have reported that the effects of voriconazole on tacrolimus levels are highly variable (Mori et al., 2012; Liran et al., 2023). Ultimately, proper dose adjustment and vigilant clinical monitoring of patients receiving this combination of treatments are mandatory.

Category × drug interactions are typically more severe, necessitating the drug combination to be avoided. The current investigation found that the number of co-administered drugs was substantially connected to the prevalence of category X pDDIs (*p* < 0.001). This outcome is consistent with the findings of earlier investigations, especially in cases requiring sophisticated therapy. A retrospective study by O. Moradi et al. found that many co-administered medications were identified as independent risk factors for pDDIs among kidney transplant recipients (Moradi et al., 2020). A notable finding in this study was identifying the APACHE Ⅱ score as an independent risk factor for category × drug interactions (*p* = 0.012). The APACHE Ⅱ score, an increasing score ranging from 0 to 71, is closely related to an increased risk of in-hospital mortality (Quintairos et al., 2023). Generally, the higher the APACHE Ⅱ score, the more serious the patient’s condition and the more heterogeneous the drug types required for treatment. Our study had no significant association of X pDDIs with other risk factors, including age, gender, BMI, primary disease, and past drug allergy. Various studies have found different factors regarding the association with the risk of pDDIs. Patients in a study conducted in a cardiothoracic intensive care unit had a strong correlation between pDDIs and age (Haitao et al., 2022). Moreover, a significant association of pDDIs was found with hospital stay duration in another study on cancer patients (Alnaim et al., 2022).

Several limitations could be considered for this research. Firstly, being a retrospective single-center study, the sampling was sequential and limited to 114 participants; thus, the patient characteristics and the treatment regimens may not be universal. Secondly, we analyzed pDDIs using a single DDI database. Consequently, it may overlook pDDIs not detected by the Lexicomp database. Furthermore, we did not gather information on the use of any drugs that were irregularly and intermittently given as needed or herbal remedies, both of which could be sources of DDIs. A significant constraint of this study was the unavailability of the partly essential data to evaluate some DDI-induced adverse outcomes.

## Conclusion

The present work identified a high frequency of potential and real DDIs in ICU lung transplant recipients after the initial transplant. Immunosuppressive drugs administered with azole exhibited a high risk of producing clinically significant interactions. The number of co-administered medications and the APACHE Ⅱ score were associated with an increased risk of category × drug interactions. This observation strengthens the need for thorough monitoring of clinical and laboratory parameters to ensure successful lung transplantation and prevent adverse drug reactions related to DDIs. Future research must adopt a multicenter prospective design with a multidisciplinary team to assess the DDIs and the clinical consequences to provide a comprehensive evaluation of DDIs.

## Data Availability

The raw data supporting the conclusion of this article will be made available by the authors, without undue reservation.
