# Supplementary material for: Prevalence and clinical significance of potential drug-drug interactions among lung transplant patients
Source: Front Pharmacol. 2024 Feb 6;15:1308260. doi: 10.3389/fphar.2024.1308260 (PMC10876870; doi:10.3389/fphar.2024.1308260)
Supplement: Supplementary file 1 [file DataSheet1.DOCX]

***Supplementary material***

**S1 Table. Unsearched drugs in the Lexicomp® Drug Interaction Software**

| **Number** | **Categories of drugs** | **Generic name** |
| --- | --- | --- |
| **1** | Digestive system drugs | Clostridium Butyricum Powders |
| **2** | Chinese patent medicine | Wuzhi Jiaonang |
| **3** | Chinese patent medicine | Jinshuibao Pian |
| **4** | Analgesics | Dezocine Injection |
| **5** | Cardiovascular system drugs | Recombinant Human Brain Natriuretic Peptide for Injection |
| **6** | Blood system drugs | Recombinant Human Thrombopoietin Injection |
| **7** | Blood system drugs | Recombinant Human Granulocyte Colony Stimulating Factor for Injection |
| **8** | Hormone drugs | Posterior Pituitary Injection |
| **9** | Vitamin drugs | Tabellae Fursultiamini |
| **10** | Digestive system drugs | Hydrotalcite Chewable Tablets |
| **11** | Digestive system drugs | Magnesium Isoglycyrrhizinate Injection |
| **12** | Digestive system drugs | Ademetionine 1,4-Butanedisulfonate for Injection |
| **13** | Digestive system drugs | Polyene Phosphatidylcholine Injection |
| **14** | Respiratory system drugs | Compound Methoxyphenamine Capsules |
| **15** | Blood system drugs | Enferal Nutritional Suspension |
| **16** | Blood system drugs | Human Interleukin-11 |
| **17** | Respiratory system drugs | Eucalyptol, Limonene and Pinene Enteric Capsules |
| **18** | Blood system drugs | Recombinant Human Erythropoientin Injection |
| **19** | Respiratory system drugs | Sivelestat Sodium for Injection |
| **20** | Nervous system drugs | Oryzanol Tablets |
| **21** | Nutritional drugs | Compound α-Ketoacid Tablets |
| **22** | Digestive system medication | Bicyclol Tablets |
| **23** | Nervous system drugs | Monosialotetrahexosy lganglioside Sodium Injection |
| **24** | Chinese patent medicine | Bailing Jiaonang |
